# Supplementary material for: P-TRAP: a Panicle Trait Phenotyping tool
Source: BMC Plant Biol. 2013 Aug 29;13:122. doi: 10.1186/1471-2229-13-122 (PMC3848748; doi:10.1186/1471-2229-13-122)
Supplement: Additional file 7 — P-TRAP source code. The Netbeans project that contains the source code of the application. [file 1471-2229-13-122-S7.zip › javadoc.zip!/javadoc/org-basmati-workspace/org/basmati/workspace/FindGrainsAction.html]

FindGrainsAction (WorkSpace)


JavaScript is disabled on your browser.


- Package
- Class
- Tree
- Deprecated
- Index
- Help

*org.basmati.workspace 1.0*

- Prev Class
- Next Class

- Frames
- No Frames

- All Classes

- Summary:
- Nested |
- Field |
- Constr |
- Method

- Detail:
- Field |
- Constr |
- Method


org.basmati.workspace

## Class FindGrainsAction

- java.lang.Object
- - org.basmati.workspace.FindGrainsAction

- All Implemented Interfaces:
  :   java.awt.event.ActionListener, java.util.EventListener

  ---

    

  ```
  public final class FindGrainsAction
  extends java.lang.Object
  implements java.awt.event.ActionListener
  ```

  The handler of finding all grains in all image in the selected project.

  Author:
  :   faroqal-tam

- - ### Constructor Summary

    Constructors

    Constructor and Description

    `FindGrainsAction(org.bioalg.riceprojecttype.RiceProject context)`
  - ### Method Summary

    Methods

    | Modifier and Type | Method and Description |
    | --- | --- |
    | `void` | `actionPerformed(java.awt.event.ActionEvent ev)` |

    - ### Methods inherited from class java.lang.Object

      `clone, equals, finalize, getClass, hashCode, notify, notifyAll, toString, wait, wait, wait`

- - ### Constructor Detail


    - #### FindGrainsAction

      ```
      public FindGrainsAction(org.bioalg.riceprojecttype.RiceProject context)
      ```
  - ### Method Detail


    - #### actionPerformed

      ```
      public void actionPerformed(java.awt.event.ActionEvent ev)
      ```

      **Specified by:**
      :   `actionPerformed` in interface `java.awt.event.ActionListener`


- Package
- Class
- Tree
- Deprecated
- Index
- Help

*org.basmati.workspace 1.0*

- Prev Class
- Next Class

- Frames
- No Frames

- All Classes

- Summary:
- Nested |
- Field |
- Constr |
- Method

- Detail:
- Field |
- Constr |
- Method
